# Food safety knowledge among pregnant women in the United Arab Emirates amid the COVID-19 pandemic

**DOI:** 10.1371/journal.pone.0279810

**Published:** 2022-12-30

**Authors:** Rameez Al Daour, Tareq M. Osaili, Mona Hashim, Ioannis N. Savvaidis, Nezar Ahmed Salim, Anas A. Al-Nabulsi, Hala Bahij ElSayegh, Nawal Hubaishi, Ayla Coussa, Anastasia Salame, Maysm N. Mohamad, Sheima T. Saleh, Hayder Hasan, Ayesha S. Al Dhaheri, Lily Stojanovska, Leila Cheikh Ismail

**Affiliations:** 1 Department of Clinical Nutrition and Dietetics, Research Institute of Medical and Health Sciences (RIMHS), College of Health Sciences, University of Sharjah, Sharjah, United Arab Emirates; 2 Department of Nutrition and Food Technology, Faculty of Agriculture, Jordan University of Science and Technology, Irbid, Jordan; 3 Dubai Hospital, Dubai Health Authority, Dubai, United Arab Emirates; 4 Warwick Medical School, University of Warwick, Coventry, United Kingdom; 5 Obstetrics and Gynecology Department, Fakih IVF Clinics, Al Ain, United Arab Emirates; 6 Department of Nutrition and Health, College of Medicine and Health Sciences, United Arab Emirates University, Al Ain, United Arab Emirates; 7 Institute for Health and Sport, Victoria University, Melbourne, Australia; 8 Nuffield Department of Women’s & Reproductive Health, University of Oxford, Oxford, United Kingdom; University of Ilorin, NIGERIA

## Abstract

Studies have indicated shortcomings in food safety knowledge and practices among pregnant women in the Arab region. A high-risk group for having severe outcomes from foodborne illnesses. This study aimed to assess self-reported food safety knowledge and practices among pregnant women in the UAE during the COVID-19 pandemic. A total of 354 pregnant women residing in the UAE completed an online survey between October 2021 and January 2022. The questionnaire included socio-demographic information, food safety knowledge, and food practices during the COVID-19 pandemic. Correct answers for food safety knowledge were scored out of 50 and the total score was compared by sociodemographic characteristics. The total mean score for the study population was 26.7 ± 4.6 out of 50. Participants had good knowledge about foodborne diseases (81.3%) and personal hygiene practices (61.8%). While they were least knowledgeable about cross-contamination (43.3%) and temperature control practices (35.8%). Significantly higher knowledge scores were observed with higher levels of education and primigravida women (*p*<0.05). Knowledge about the COVID-19 virus and its relation to food safety was adequate for most participants. This study infers the need for food safety-related education and training programs to reduce the risk of foodborne disease among this vulnerable group. It also highlights the need to enhance the role of healthcare professionals as trusted sources of information in improving food safety during pregnancy.

## Introduction

In early 2020, the World Health Organization declared COVID-19 to be a global pandemic [1]. Acknowledging the risks and in efforts to break the virus transmission chain, authorities implemented measures such as social distancing, use of face masks, and movement restrictions as well as provided recommendations to sustain adequate food supply, food safety, and nutritional status of the population [2]. Retrospective investigation of COVID-19 positive cases indicated that virus virulence, individual susceptibility, and immunity status are considered key determinants of exposure to the COVID-19 disease [3]. With that, evidence showed that pregnant women are more susceptible to severe complications when infected with COVID-19 as opposed to their non-pregnant counterparts. Further, while adverse outcomes such as preterm birth and cesarean deliveries are consistently reported for infected pregnant women, actual mother-to-fetus vertical transmission of the virus remains controversial to date [4].

The measures and restrictions implemented towards containing the spread of COVID-19 inevitably contributed to increased food preparation and consumption at home [5]. The domestic environment can be considered a high-risk environment for foodborne diseases [6], given that no guidelines are vigorously implemented in home settings compared to restaurants and other food establishments. Foodborne diseases represent a serious public health issue having great impacts on human health and health care systems worldwide [7]. According to the latest estimates, almost 1 in 10 people mounting to 600 million people worldwide fall ill each year due to the consumption of contaminated food or water [8]. In particular, pregnant women are a high-risk group for falling ill from foodborne diseases due to several immunological alterations affecting their susceptibility to communicable diseases [9]. Hence, it is highly advised that this group avoid high-risk food consumption and ensure proper handling and preparation of food.

Pregnancy is a time when women become highly driven to educate themselves about food safety due to their increased concern about their health and their unborn child [10]. Education during pregnancy regarding food safety and emphasis on the consumption of low-risk foods is crucial to preventing foodborne disease [11]. In the United Arab Emirates (UAE), food safety authorities and municipalities play a pivotal role in ensuring food safety in the country and promoting messages and advice to the public. In this particular group, previous international research identified gaps in knowledge and practices of basic food safety aspects in food preparation, temperature control, cross-contamination, safe food consumption, and personal hygiene [10, 12, 13]. Moreover, a recent study in the UAE found that non-pregnant women had an acceptable level of knowledge on food safety regarding cross-contamination and foodborne diseases but lacked detailed knowledge on risk factors for foodborne diseases and food preparation and consumption [14].

The incidence of foodborne diseases is associated with sheer insufficiency of knowledge and subsequent inadequacy of attitudes regarding proper food safety practices in home settings [15]. Consequently, previous studies have shown that consumers’ proper knowledge of food safety is vital to dwindling the risk of foodborne diseases during food preparation at home [14]. Given the ongoing COVID-19 pandemic, consumer concerns related to food safety have progressed from falling ill from foodborne diseases to fear of being infected with the COVID-19 virus from food and food packages [16]. Inevitably, the impact of this unprecedented global pandemic has been manifested in most countries worldwide affecting eating habits, physical activity, mental health, food safety knowledge as well as shopping and hygiene practices [17].

Although a few studies focusing on food safety knowledge and practices among pregnant and non-pregnant women are available in the literature, studies on this vulnerable group in the Arab region and the UAE are lacking. Therefore, acknowledging the importance of assessing food safety knowledge and establishing integrated food safety programs is vital to diminishing risk factors that lead to foodborne disease.

This study aims to assess the level of awareness of basic food safety and handling practices among pregnant women in the UAE during the COVID-19 pandemic.

## Materials and methods

### Study design and participants

This is a descriptive cross-sectional quantitative study conducted between October 2021 and January 2022 to assess the level of food safety knowledge among pregnant women living in the UAE amid the COVID-19 pandemic. The inclusion criteria were pregnant women who are 18 years or older and residing in the UAE. Participants were recruited from public and private antenatal and obstetrics outpatient clinics in the UAE through convenient sampling. The clinics included one public hospital in Sharjah, a public hospital and a private clinic in Dubai, two private clinics in Abu Dhabi, and a private clinic in Al Ain. Trained researchers approached pregnant women in the waiting area and provided a brief introduction and explanation about the study. Those who agreed to take part in the study signed a written informed consent form. Participants were then sent a web link through a text message (SMS), or via WhatsApp™ to complete the survey. Based on the participants’ request, some women completed a printout of the survey. An information sheet was provided at the beginning of the survey describing the study protocol and objectives. Participants were informed that they can exit the survey or withdraw at any point and that all data was collected anonymously.

The minimum number of necessary sample size was calculated based on the following formula to be 345:

$$n=\frac{Z^{2}\times P\times (1-P)}{E^{2}}$$

The proportion of the population was established from previous research findings among non-pregnant females in the UAE [14]. The confidence level was set at 95% with 5% error margin. Out of 398 women invited to participate in the study, a total of 354 pregnant women completed the questionnaire and were included in the data analyses (response rate = 89%).

The study protocol obtained ethical approval from the Research Ethics Committee at the University of Sharjah (reference number: REC-21-01-12-01), the Ministry of Health and Prevention Research Ethics Committee (reference number: MOHAP/DXB-REC/FMM/NO. 13/2021), and the Dubai Scientific Research Ethics Committee of the Dubai Health Authority (reference number: DSREC-04/2021_07). A written informed consent was obtained from all participants.

### Survey questionnaire

The questionnaire development, translation, and content were described in a previous publication [18]. Some additional questions were added to investigate the effect of the COVID-19 pandemic on food safety practices. Content validity has been established by experts in the field of nutrition and food safety. The tool showed good content validity between levels of expertise. Test-retest reliability was established by 10 pregnant women. Internal consistency reliability was established with items personal hygiene (alpha r> 7.23), cross-contamination (alpha r> 7.01), temperature control (alpha r> 7.89), food consumption and safety (alpha r> 8.02), cleaning and sanitation (alpha r> 7.69), knowledge of foodborne diseases (alpha r> 7.62) and COVID-19 questions (alpha r> 8.31). The pilot-testing data was not included in the results of this research. The final version of the questionnaire was either printed or developed using Google Forms and a Uniform Resource Locator (URL) was distributed to participants.

The questions of the survey are divided into three main sections: (1) socio-demographic data (age, education level, household income, emirate of residence, number of pregnancies, trimester of pregnancy, and if food safety education was provided during pregnancy); (2) food safety awareness questions (personal hygiene, cross-contamination, temperature control, food consumption and safety, cleaning and sanitation, and foodborne diseases); (3) attitudes and practices toward food safety during the COVID-19 pandemic. The full questionnaire is provided as a S1 File.

### Statistical analysis

Printout responses were entered into the online form and all responses were exported from Google forms as an Excel file where data was cleaned and coded and then imported into the Statistical Package for the Social Sciences (SPSS) ver. 26.0 (IBM, Chicago, IL, USA) for analysis. Counts and percentages were used to report descriptive statistics for the sociodemographic characteristics. Sub-scores for each test aspect (i.e., personal hygiene, cleaning, and sanitation, cross-contamination, temperature control, food consumption and safety, knowledge of foodborne diseases) were calculated by summing the correct responses. Correct responses were based on the recommendations of the WHO in the “five keys to safer food manual” [19] and the information in the “food safety booklet for pregnant women, their unborn babies, and children under five” by the FDA [20]. A total score out of 50 was derived for each participant determining food safety knowledge based on the sum of scores from all aspects. Mean responses and percentages of responses in each category were computed. Student’s t-test and Analysis of Variance (ANOVA) were used to compare the mean sum of scores of every knowledge section by demographic variables. Results were significant at *p* < 0.05.

## Results

### Participants characteristics

A total of 354 participants were included in the study. Table 1 presents the general characteristics of the study participants. Overall, most of the participants were in the age range of 29 to 39 years (65.8%), were local citizens (52.7%), and had either completed a college education or above (59.6%). Over half of the participants were in their last trimester (55.6%). About 56% reported receiving food safety information during pregnancy. The main reported sources of information were doctors and the internet/social media (31.6% and 26.6%, respectively).

**Table 1:** Sociodemographic characteristics of the study participants (n = 354).

Characteristic	*n*	(%)
**Age (year)**		
18–28	91	25.7
29–39	233	65.8
40 and above	30	8.5
**Nationality** ^**a**^		
Local citizens	184	52.7
Residents	165	47.3
**Educational level**		
Less than high school	30	8.5
High school	113	31.9
College or above	211	59.6
**Emirate of residence**		
Abu Dhabi	97	27.4
Dubai	120	33.9
Sharjah	73	20.6
Northern Emirates ^b^	64	18.1
**Number of pregnancies**		
First pregnancy	116	32.8
Second pregnancy and above	238	67.2
**Trimester**		
First trimester	85	24.0
Second trimester	72	20.3
Third trimester	197	55.6
**Received any food safety information during pregnancy**		
Yes	199	56.2
No	155	43.8
**Sources of food safety information (n = 199)** ^c^		
Doctors	112	31.6
Nurses	30	8.5
Nutritionist	60	16.9
Internet/social media	94	26.6
Brochures	20	5.6
Family and friends	51	14.4
Television	15	4.2

^a^ Data missing

^b^ Northern emirates (Ajman, Umm Al Quwain, Ras Al Khaimah, Fujairah)

^c^Multiple answers were allowed

### Food safety and risk perception

**Fig 1:**
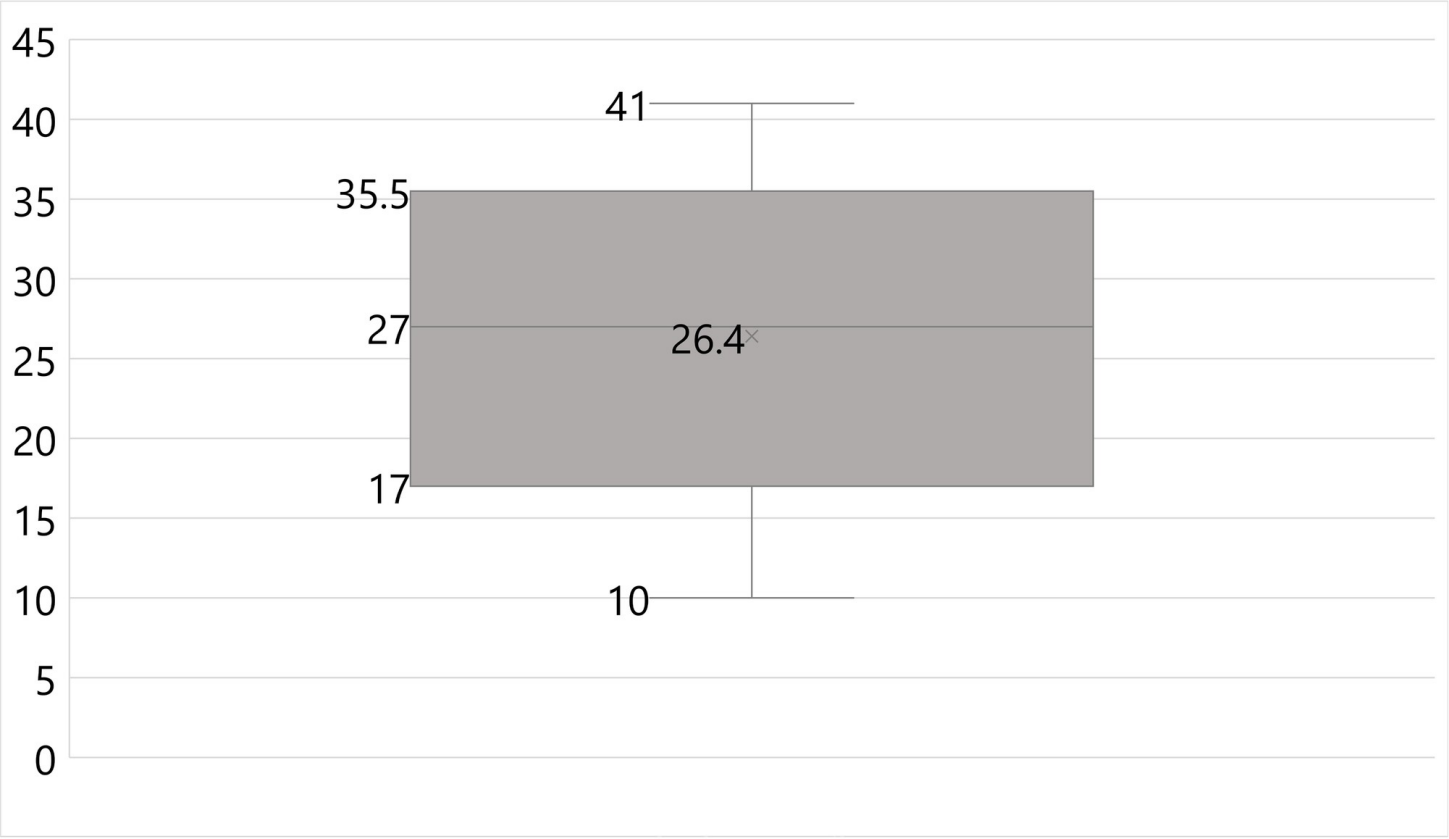
Food safety knowledge among participants (n = 354).

The mean score was calculated for each participant based on the totality of correct responses. The total mean score for the study population was 26.7 ± 4.6 with a score percentage of 53.4% as shown in Table 2. The range of total food safety mean scores is portrayed in a box and whisker plot as shown in Fig 1. Participants were most knowledgeable about foodborne diseases followed by personal hygiene aspects (score percentage of 81.3% and 61.8% respectively). Participants were least knowledgeable about cross-contamination and temperature control aspects (score percentage of 43.3% and 35.8% respectively). Regarding food consumption and safety and cleaning and sanitation aspects, participants were able to answer only half of the questions correctly (score percentages of 51.1% and 50.0% respectively).

**Table 2:** Food safety score for study participants (n = 354).

Domains	Mean ± SD	% of the score
Personal Hygiene (out of 11)	6.8 ± 1.6	61.8
Cross Contamination (out of 6)	2.6 ± 1.1	43.3
Temperature Control (out of 12)	4.3 ± 1.7	35.8
Food consumption and safety (out of 9)	4.6 ± 2.0	51.1
Cleaning and sanitation (out of 4)	2.0 ± 0.8	50.0
Knowledge of foodborne diseases (out of 8)	6.5 ± 1.3	81.3
**Total score for food safety awareness (Range 0–50)**	**26.7 ± 4.6**	**53.4**

### Personal hygiene

The food safety knowledge score for the personal hygiene aspect was 6.8 ± 1.6 (out of 11) corresponding to 61.8% as shown in Table 2. Moreover, the query statements and participants’ correct responses to personal hygiene questions are presented in Table 3. Most participants were aware of the importance of washing hands before food preparation, after touching raw meat or raw eggs, after coughing or sneezing, and after waste disposal (83.6% - 96.3%). Moreover, a far less proportion was knowledgeable about the importance of wearing gloves during cooking (17.2%) and only two participants corresponding to less than 1% knew that it is not necessary to wash their hands during salad preparation.

**Table 3:** Query statements and responses^1^ to the “personal hygiene” aspect (n = 354).

Query statements	n (%)
1. Wash hands well before starting to prepare or cook food	325 (91.8)
2. Wash hands well after touching any part of the body during food preparation	236 (66.7)
3. Wash hands well after touching raw meat	341 (96.3)
4. Wash hands well after touching raw eggs	313 (88.4)
5. Not necessary to wash hands well during salad preparation	2 (0.6)
6. Wash hands well after coughing and sneezing during food preparation or cooking	296 (83.6)
7. Wash hands well after disposing of waste	326 (92.1)
8. Time to spend on washing hands ≥ 20 s	65 (18.4)
9. Wash hands the correct way ‘Wet hands with running warm water, use soap and then wash with running warm water, wipe dry’	214 (60.5)
10. Wear gloves during food preparation or cooking	61 (17.2)
11. Cover or tie hair during food preparation or cooking	225 (63.6)

^**1**^ Correct response

### Cross-contamination

A suboptimal knowledge score with regards to the cross-contamination aspect among our participants was observed (2.6 ± 1.1; 43.3%) as shown in Table 2. The query statements and participants’ correct responses are presented in Table 4. Only one in four pregnant women knew how to properly wash and disinfect a knife used previously for cutting raw meat to cut vegetables and less than one in five of them correctly identified that using a spoon used in cooking for tasting as a high-risk practice (25.7% and 16.1% respectively).

**Table 4:** Query statements and responses^1^ of “cross-contamination prevention” aspect (n = 354).

Query statements	n (%)
1. Wash knife used to cut raw meat or poultry with water, soap and use disinfectant before using it to chop vegetables	91 (25.7)
2. Store vegetable salad above raw meat or chicken in the refrigerator	112 (31.6)
3. Store raw eggs separately (protected) from other foodstuffs in a refrigerator	198 (55.9)
4. Using the same spoon used to stir the food to taste the food during cooking is the least safe way	57 (16.1)
5. COVID-19 virus cannot be transmitted by the food	188 (53.1)
6. COVID-19 virus can survive on hard surfaces for days	263 (74.3)

^**1**^ Correct response

Storing food in organized compartments in the refrigerator as per the type of food and not as per space availability plays an important role in preventing cross-contamination [21]. However, pregnant women in the present study were not aware of that. This was evident as about half of the participants correctly identified that they should store eggs separately from other food and only a third of them knew that vegetables should be stored above raw meat or poultry (55.9% and 31.6% respectively). These findings are lower than the study among women in Dubai (64.25%) [22] and those of women in Saudi Arabia (63.0%) [23].

Two questions in this section tackled the current COVID-19 pandemic and tested pregnant women’s knowledge about its relation to cross-contamination. Around three in four participants knew that the COVID-19 virus can persist on hard surfaces for multiple days (74.3%), Whereas only half of them knew that it cannot be transmitted by food (53.1%). To date, no evidence has associated the transmission of the COVID-19 virus with food handling or food consumption [2]. However, it is suggested that food or food packages can be carriers of the virus [24] and when contaminated people in contact may get infected when touching their nose, mouth, or eyes [2].

### Temperature control

Knowledge with regards to the temperature control aspect among our participants was the most lacking as the lowest mean score was recorded (4.3 ± 1.7; 35.8%) as shown in Table 2. The query statements and participants’ correct responses are presented in Table 5. Most participants knew that frozen items should be purchased toward the end of shopping, leftovers should be discarded after two days of preparation and storage, and that reheated leftovers should be discarded immediately (77.1%, 75.4% and 58.2%, respectively). However, 36.7% knew that they had to store leftovers immediately in the fridge and reheat when needed, and only 22.6% knew the safest way to reheat leftovers (until it boils). A low proportion of the participants knew the accurate storing temperature of chilled ready-to-eat foods, frozen foods, and hot ready-to-eat foods (21.8%, 15.5%, and 2.3% respectively).

**Table 5:** Query statements and responses^1^ of the “temperature control” aspect (n = 354).

Query statements	n (%)
1. Defrost frozen raw meat or chicken in the refrigerator	72 (20.3)
2. Reheat leftover until it boils	80 (22.6)
3. Meat or poultry look cooked by their temperature	26 (7.3)
4. Put prepared food in the refrigerator, then reheat when ready to eat	130 (36.7)
5. Discard reheated leftovers immediately	206 (58.2)
6. Leftovers should be kept in the fridge for no more than 2 days	267 (75.4)
7. Purchase frozen food at the end of shopping time	273 (77.1)
8. Store chilled ready-to-eat foods at 1 to 4°C	77 (21.8)
9. Store frozen foods at -18°C	55 (15.5)
10. Store hot ready-to-eat foods at >60°C	8 (2.3)
11. Cook chicken till it reaches 73°C	59 (16.7)
12. COVID-19 virus cannot multiply in the food	260 (73.0)

^**1**^ Correct response

Within this section, one question addressed the current COVID-19 pandemic and tested pregnant women’s knowledge about its relation to temperature control. Our participants were aware that the COVID-19 virus cannot multiply in food (73.0%) which is backed up by scientific evidence as viruses cannot multiply without a human or animal host [2].

### Food consumption and safety

The food safety awareness score for the food consumption and safety aspect was 4.6 ± 2.0 corresponding to 51.1% as shown in Table 2. In the present study, we asked the participants about their consumption frequency of some high-risk food items as shown in Table 6. The most reported high-risk foods consumed among our participants which is reflected by the least proportion of participants reporting ‘never consuming’ included white cheeses, Shawarma, desserts containing raw eggs, and semi-cooked eggs with runny yolks (25.4%, 26.6%, 44.9%, and 56.2% respectively). Moreover, less than two-thirds of the participants reported never consuming cold deli meat (64.1%) and hot dogs (64.7%) while most of them never consume raw fish (79.4%) and unwashed fruits and vegetables (87.6%). Our participants were also asked how they can tell if a food is contaminated and less than 10% of them correctly answered that it cannot be determined from appearance or taste.

**Table 6:** Query statements and responses^1^ to the “food consumption and safety” aspect (n = 354).

Query statements	n (%)
1. Never eat fried or boiled eggs with running yolks	199 (56.2)
2. Never eat pastries and cakes containing raw eggs	159 (44.9)
3. Never eat cold deli meat	227 (64.1)
4. Never eat white cheeses (Nabulsi cheese, Halloumi, etc.)	90 (25.4)
5. Never eat hot dogs	229 (64.7)
6. Never eat Shawarma	94 (26.6)
7. Never eat raw fish (e.g., sushi)	281 (79.4)
8. Never eat fruits and vegetables without washing	310 (87.6)
9. Food contaminated with pathogenic bacteria cannot be detected from its appearance or taste	35 (9.9)

^1^ Correct response

### Cleaning and sanitation

Table 7 shows the query statements and participants’ correct responses for the cleaning and sanitation aspect. Overall, the participants lacked sufficient knowledge about cleaning and sanitation which corresponded to a score of 2.0 ± 0.8 and a score percentage of 50.0% as shown in Table 2. Participants knew the proper way of washing kitchen countertop (87.6%), but had lower knowledge of the proper way to wash fresh fruits and vegetables (9.6%).

**Table 7:** Query statements and responses^1^ to the “cleaning and sanitation” aspect (n = 354).

Query statements	n (%)
1. Wash fresh fruits and vegetables with cold running water to avoid infections	34 (9.6)
2. Wash kitchen countertop with cleaning material, then wash it with water, then wipe with disinfectant	310 (87.6)
3. Sanitize the kitchen sink drain daily	270 (76.3)
4. The least safe method to disinfect the kitchen sponge is by soaking and washing in water	98 (27.7)

^1^ Correct response

### Foodborne diseases

The food safety awareness score for the foodborne disease aspect was 6.5 ± 1.3 corresponding to 81.3% as shown in Table 2. This aspect received the highest score of all aspects in the present study suggesting relatively adequate knowledge among pregnant women about foodborne diseases. Query statements and responses to the knowledge of foodborne diseases questions are shown in Table 8.

**Table 8:** Query statements and responses^1^ to the “knowledge of foodborne diseases” aspect (n = 354).

Query statements	n (%)
1. Food poisoning is more serious for pregnant women	306 (86.4)
2. Diarrhea and vomiting are symptoms of food poisoning	345 (97.5)
3. Abdominal pain and cramps are symptoms of food poisoning	320 (90.4)
4. Hair falling is not a symptom of food poisoning	321 (90.7)
5. Hypertension is not a symptom of food poisoning	251 (70.9)
6. A drop in Blood Sugar is not a symptom of food poisoning	233 (65.8)
7. Cold and Cough are not symptoms of food poisoning	258 (72.9)
8. Pregnant women are at higher risk of complications than other healthy adults if they get COVID-19	260 (73.4)

^1^ Correct response

In light of the COVID-19 pandemic, pregnant women were identified as a high-risk group to develop more severe symptoms when infected compared to the others [4]. This was a known fact by almost three-quarters of our participants who knew that pregnant women are at higher risk of complications than other healthy adults if they get COVID-19 (73.4%).

### Association between food safety knowledge and demographic characteristics

The relationship between the total food safety awareness score and selected demographic characteristics is shown in Table 9. A statistically significant difference was observed between the total score and the level of education (*p* = 0.016), where higher scores were obtained for participants with diploma degrees or above. Moreover, higher food safety awareness scores were significantly associated with lower parity (*p*<0.001) as participants experiencing their first pregnancy were more knowledgeable as opposed to women experiencing their second pregnancy or above.

**Table 9:** Association between total food safety awareness score sociodemographic characteristics of participants (n = 354) ^1^.

Characteristics	Knowledge score Mean ± SD	*P*-value
**Age (Year)**		0.118
18–28	27.2 ± 4.7	
29–39	26.7 ± 4.6	
40 and above	25.2 ± 4.8	
**Educational level**		**0.016**
Less than high school	25.3 ± 5.3	
High school	26.6 ± 5.1	
College or above	27.4 ± 4.3	
**Number of pregnancies**		**<0.001**
First pregnancy	28.1 ± 4.4	
Second pregnancy or above	26.1 ± 4.6	

^1^ Analysis of Variance (ANOVA) was conducted to compare the mean sum of correct responses of every section (knowledge score) by demographic variables

### Food safety and COVID-19

**Fig 2:**
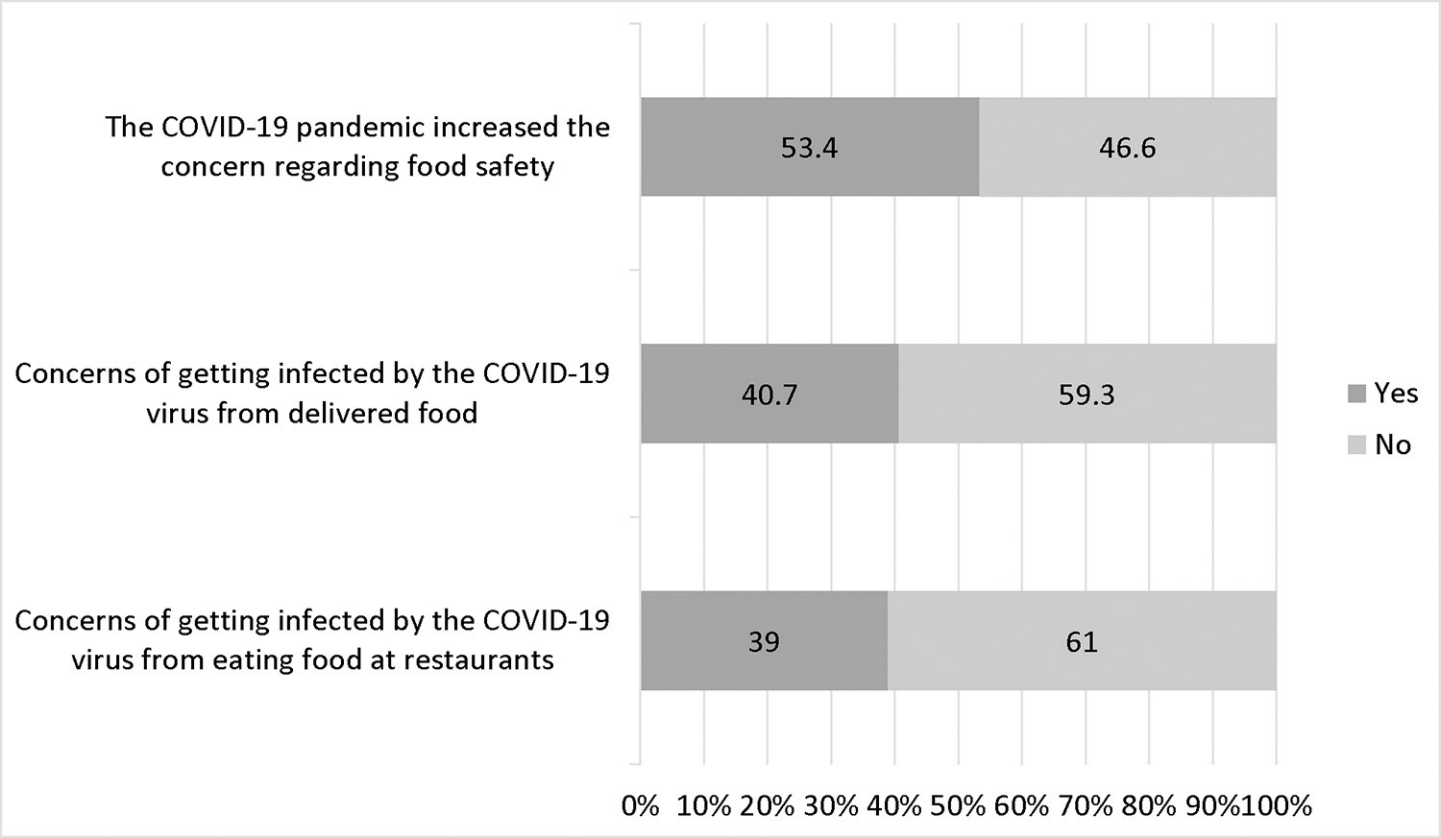
Attitude toward food safety and COVID-19 (n = 325).

Fig 2 illustrates the participants’ attitudes toward food safety considering the COVID-19 pandemic. As the figure shows, around half of the participants reported an increased level of concern regarding food safety due to the COVID-19 pandemic (53.4%). Moreover, less than half of the participants expressed concerns about getting infected by the COVID-19 virus from delivered food while a third of them were concerned about getting infected from eating out (40.7% and 39.0% respectively). Although no evidence suggests the transmission of COVID-19 through food or food packaging, several recommendations are in play regarding food safety among food handlers, producers, and consumers as precautionary measures during the pandemic [2].

**Fig 3:**
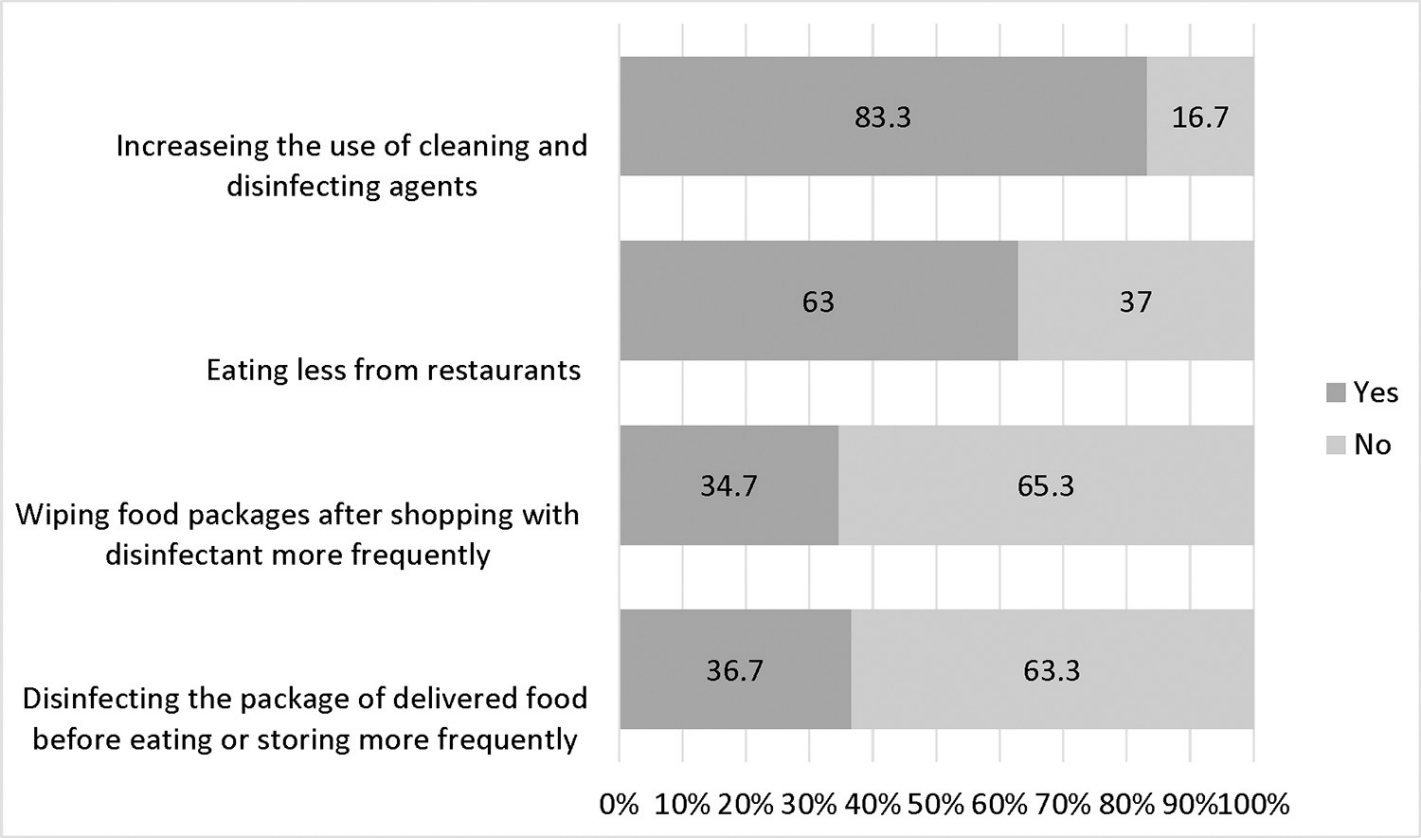
Practices during the COVID-19 (n = 325).

**Fig 4:**
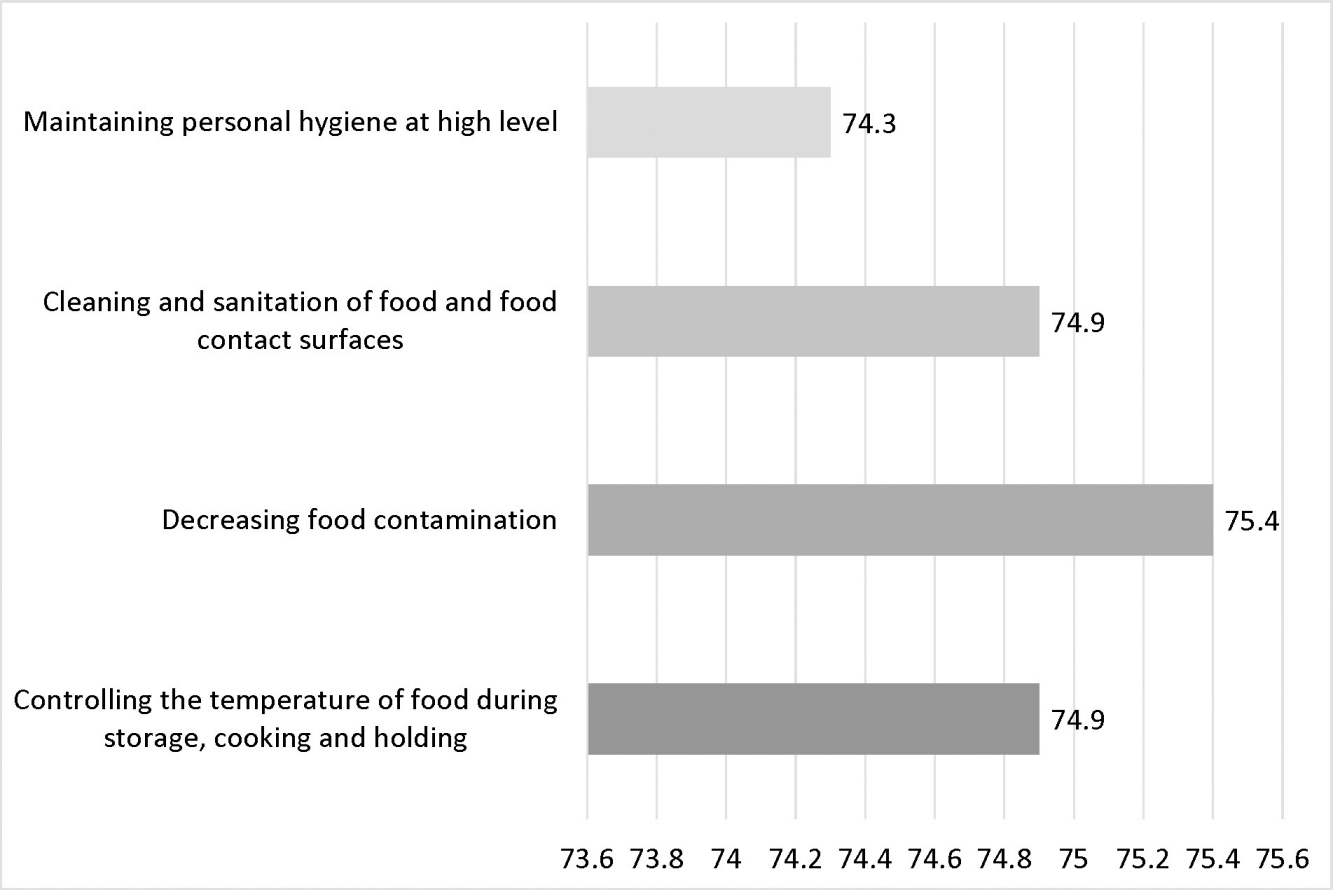
Participants who answered ’Yes’ to “Did the COVID-19 pandemic enhance your food safety measures in the following aspects?” (n = 325).

The reported practices of the participants during the COVID-19 pandemic are depicted in Fig 3. Most of the participants reported increased use of cleaning and disinfecting agents amid the pandemic and about two-thirds of them reported eating out less frequently (83.3% and 63.0% respectively). On the other hand, about a third of the participants reported wiping purchased food packages and delivering food before eating or storing (34.7% and 36.7% respectively). Sustaining proper personal hygiene, frequent cleaning, and regular disinfection of surfaces are all key recommendations that control the transmission of the COVID-19 virus [25]. COVID-19 had a positive impact on enhancing food safety measures among our participants as shown in Fig 4. This was manifested by around three-quarters of the participants agreeing that the pandemic enhanced the measures taken to decrease food contamination, maintain personal hygiene at a high level, control the temperature of food during storage, cooking, and holding, and clean and sanitize food and food contact surfaces.

## Discussion

The present study identified certain gaps in knowledge in food safety-related aspects among pregnant women in the UAE. In the present study, the total food safety awareness score was about 26 out of 50 (53.4%) which was similar to that of non-pregnant women in Dubai (53.3%) [22] and lower than that of women in Sharjah (57.4%) [14]. In contrast to the women population in other Arab countries, the knowledge score was higher than that of female college students in Jordan (41.3%) [26] and lower than that of women in Egypt (67.4%) [27].

In this study, only one half of the participants reported receiving food safety information during their pregnancy and of those, doctors were ranked first as a food safety information source. Healthcare professionals are usually considered the preferred source of food safety-related information [28], yet a possible factor here might be inconsistency of the advice delivered to patients or how well the patients comprehend the information received. Moreover, social media and the internet ranked second in the sources of information. Research on the influence of social media on consumers’ food safety knowledge indicates a significant relationship between source credibility and the content of comments [29]. As such negative comments were associated with more negative responses regardless of the credibility of the source. Thus, it is crucial to encourage pregnant women to obtain information from credible and valid sources.

Hands could be a chief vehicle for spreading pathogenic microbes in the kitchen and the importance of proper hand washing lies in its role in avoiding cross-contamination [30]. Moreover, maintaining personal hygiene in all stages of food preparation is key to preventing and controlling the occurrence of foodborne [31]. In our study pregnant women were able to identify most cases in which hand washing is necessary. However, disparities in knowledge can be observed when participants were asked about the proper way to wash their hands and the time required to spend washing them (>20 seconds). This corresponds to less than two-thirds of the participants correctly identifying the proper method of handwashing and less than one fifth of them being aware of the proper duration of handwashing. It is well established that washing hands for at least 20 seconds is crucial in averting foodborne disease [32], and that the importance of proper handwashing surpasses the preventative effects of wearing gloves during cooking [33]. Our findings regarding the proper method of handwashing are lower than that of pregnant women in Slovenia (rated 4.28 out of 5 in the scale of importance) [15], mothers in Saudi Arabia (95.8%) [34], women in the UAE (54%) [22], and female college students in Jordan (51%) [26].

Participants in this study had low knowledge about cross-contamination practices like using different cutting boards for meat and vegetable. A review paper on food safety in home kitchens indicated that almost 14% of all foodborne disease cases in the UK are attributed to improperly cleaned cutting boards and knives and that the majority of consumers in home settings do not properly clean them to avert cross-contamination [30]. In contrast to our findings, a study among women in Jordan reported that a high proportion of participants (61.6%) who use separate sets of cutting boards and knives for meat or vegetable and who wash them after each use [26]. Moreover, pregnant women in Slovenia were found to be more concerned with using separate cutting boards and knife sets as opposed to non-pregnant women [13].

More than three-quarters of the participants knew that frozen items should be purchased toward the end of shopping time. This agrees with findings reported in the UAE (64%) [14] and Saudi Arabia (73.6%) [34]. The significance of this practice lies in the fact that it decreases the duration of exposure of frozen food to lower than freezer optimal temperatures and prevents rapid bacterial growth [35]. However, a substantially lower proportion knew the safest way to reheat leftovers; that is ‘until it boils’ (22.6%) which was similar to that of Saudi mothers (31.6%) [34] and lower than women in Dubai (50.1%) and Slovenia (60.0%) [22, 36]. Moreover, around a third of our participants were aware of storing leftovers immediately in the fridge and reheating when needed (36.7%). This was lower than the findings from Dubai (71.6%) and Saudi Arabia (71.0%) [22, 23].

Temperature control is an integral aspect of food safety as sufficient knowledge and subsequent proper practices play a substantial role in foodborne disease prevention. In the present study, only one in five pregnant women correctly identified thawing frozen meat or poultry in the refrigerator as the most proper method of defrosting. Studies among pregnant women in Turkey, mothers in Saudi Arabia, and women in the UAE indicated quite similar results [22, 34, 37]. These findings raise concerns as improper thawing of frozen food leads to keeping the food within the temperature danger zones which increases the likelihood of bacterial growth [38].

Participants in the present study had poor knowledge concerning appropriate cooking and storing temperatures. The lowest proportion of the participants knew the accurate storing temperature of chilled ready-to-eat foods, frozen foods, and hot ready-to-eat foods. Other alarming findings were that our participants lacked knowledge of the appropriate cooking temperature of food and that the temperature of food is an indication of its doneness. Numerous studies highlighted low levels of knowledge about appropriate cooking temperatures. Moreover, they indicated that temperature abuse was the most prominent among pregnant women who did not own thermometers at home or who did not know how to use them properly [10, 12, 15, 37].

Because of the physiological alterations and changes in immunity during pregnancy, pregnant women are more prone to foodborne diseases than non-pregnant women. Therefore, while optimal nutrition is highly advocated for this vulnerable group, avoidance of high-risk foods is of great importance during pregnancy [39]. Participants in the current study did not adherence to safe food consumption by avoiding high risk foods which was consistent with findings of other studies [39, 40]. In a study comparing pregnant to non-pregnant women’s food consumption, pregnant women were less likely to prepare high-risk foods but more likely to prepare eggs with runny yolks and raw meats [15]. In another study, pregnant women were more likely to consume hot dogs or deli meats without cooking to appropriate temperatures [41]. The variability in high-risk food consumption is expected and may reflect gaps in knowledge of food safety guidelines in general in addition to specific guidelines during pregnancy. There is a myriad of disease-causing microorganisms and microbial infection may occur at any point in the food chain. While some microbes may change the quality of food (i.e. taste, odor, appearance), many pathogenic bacteria do not cause any visible changes [42] and hence, targeted education on the manifestation of pathogens in food is required.

The majority of the participants in this study were aware of the correct method of cleaning kitchen countertops which was higher than that of non-pregnant women in the UAE [14, 22] and mothers in Saudi Arabia [34]. On the other hand, less than a third of the participants correctly identified the least safe method of cleaning the kitchen sponge. To avoid foodborne diseases, kitchen sponges need to be cleaned frequently and substituted with fresh ones regularly. Overused and dirty kitchen sponges encompass many pathogenic bacteria that are known to cause foodborne diseases such as *Campylobacter spp*., *Enterobacter cloacae*, *E*. *coli*, *Salmonella spp*., *and Staphylococcus spp*, to name a few [43]. According to food safety guidelines, fresh fruits and vegetables should be washed under cold running water to demean the presence of any contaminant on their surface [44]. The present study suggested a huge deficit in awareness as less than 10% of the participants knew the correct method of washing fruits and vegetables which is relatively lower than findings among pregnant women in other studies [14, 34].

The countries in the Eastern Mediterranean region rank third in the estimated foodborne diseases per capita [45] and pregnant women are in particular 18 times more prone to infection than the general population [46]. Our findings revealed that most pregnant women were aware of the seriousness of food poisoning to their health. By contrast, research among pregnant women in the Netherlands indicated that they lack awareness of listeriosis and toxoplasmosis and prevention strategies during pregnancy [47]. Moreover, most of our participants correctly identified diarrhea and abdominal pain as symptoms of food poisoning (97.5% and 90.4%) and that hair falling, hypertension, low blood sugar, and cold and cough are not common symptoms of foodborne diseases (65.85%-90.7%). The findings are like those of non-pregnant women in the UAE, Saudi Arabia, and Jordan [14, 23, 26].

Findings of this study showed significantly higher knowledge scores with higher levels of education and primigravida women. This is in line with other studies associating higher educational levels with better food safety-related knowledge [14, 23, 34]. Moreover, a study among pregnant women in New Zealand showed significantly higher adherence to food safety guidelines among participants with lower parity [39]. This suggests that first time mothers might be more concerned about their health and the health of their infant.

### Strengths and limitations

The present study has several limitations. Firstly, the cross-sectional design does not allow to infer causality. Secondly, the use of a self-reported questionnaire may trigger respondent bias and misreporting of data. Lastly, convenience sampling in the present study may introduce selection bias. Nonetheless, the use of both online and printout surveys ensured a wide range of distribution and a higher level of convenience for the participants. Moreover, the present study is the first of its kind in the UAE providing unique insights into the level of food safety knowledge among pregnant women.

## Conclusions

Overall, our findings revealed that pregnant women in the UAE have less than optimal awareness of food safety. Our participants were least aware of temperature control and risks of cross-contamination while exhibiting more awareness concerning personal hygiene and knowledge of foodborne diseases. Moreover, the results of this study revealed that higher education and experiencing the first pregnancy were associated with overall higher knowledge scores. Further, general knowledge about the COVID-19 virus and its relation to food safety was adequate for most participants. The data obtained from this study infers the crucial need for a profound focus on food safety-related education to reduce the incidence of foodborne disease among this vulnerable group. Future research should focus on assessing the food safety practices of this group and conducting interventional studies to enhance the level of awareness and fill the major gaps of knowledge identified in the study.

## Supporting information

S1 File Study questionnaire. (PDF)
