# Correction: Food safety knowledge among pregnant women in the United Arab Emirates amid the COVID-19 pandemic

**DOI:** 10.1371/journal.pone.0314641

**Published:** 2024-11-22

**Authors:** Rameez Al Daour, Tareq M. Osaili, Mona Hashim, Ioannis N. Savvaidis, Nezar Ahmed Salim, Anas A. Al-Nabulsi, Hala Bahij ElSayegh, Nawal Hubaishi, Ayla Coussa, Anastasia Salame, Maysm N. Mohamad, Sheima T. Saleh, Hayder Hasan, Ayesha S. Al Dhaheri, Lily Stojanovska, Leila Cheikh Ismail

The affiliation for the first and fourth authors is incorrect. The correct affiliation is not indicated. Rameez Al Daour and Ioannis N. Savvaidis are not affiliated with #1 but with: Department of Environmental Health Sciences, College of Health Sciences, University of Sharjah, Sharjah, United Arab Emirates.
