# Supplementary material for: Food safety knowledge among pregnant women in the United Arab Emirates amid the COVID-19 pandemic
Source: PLoS One. 2022 Dec 30;17(12):e0279810. doi: 10.1371/journal.pone.0279810 (PMC9803218; doi:10.1371/journal.pone.0279810)
Supplement: S1 File — (PDF) [file pone.0279810.s001.pdf]

Food Safety Knowledge among pregnant women in the United Arab Emirates during the COVID-19 Pandemic

You are invited to participate in a web-based online survey entitled "Food Safety Knowledge among pregnant women in the United Arab Emirates during the COVID-19 Pandemic". This research project is conducted by the Dubai Health Authority.

Your participation in this study is completely voluntary. You may refuse to take part in the research or exit the survey at any time. The researchers will maintain total confidentiality of your responses, and all data will be anonymous without any indication of personal identity. Your responses will help us investigate and identify areas that lack health awareness regarding food safety for pregnant women during COVID-19 pandemic period.

General information to the respondents:

- You are kindly requested to participate in this research study.
- You should understand what the research is about and what it will involve before agreeing to participate.
- You have the absolute freedom to ask the researchers for more information and explanation.
- You have the absolute freedom to withdraw from the study.

Duration:

10-15 minutes to fill the online questionnaire.

Risks:

No risk is there from filling the online Questionnaire.

Consent:

• I have read and I understand the provided information and have had the opportunity to ask questions.	☐
• I understand that my participation is voluntary and that I am free to withdraw at any time, without giving a reason and without cost.	☐
• I understand that I will be given a copy of this consent form.	☐
• I voluntarily agree to take part in this study.	☐

If you have any questions or would like a copy of this consent letter, please contact:

- Mr. Nezar Salim, Researcher and Nurse Educator, Dubai Hospital. Email: nsalim@dha.gov.ae
- Dr Nawal Hubaishi, Consultant of obstetrics and Gynecology, Dubai Hospital. Email: nmhubaishi@dha.gov.ae
- Ms. Hala elsayegh, Head of Gynecology nursing, Dubai Hospital. Email: hbelsayegh@dha.gov.ae

Part 1: Attitude and Perception

This part is designed to assess your attitude and perception toward food safety during the COVID-19 pandemic. Please indicate how often you practice the following from Always to never:

Personal Hygiene

1. Do you wash your hands well before starting with food preparation or cooking?
   - ☐ Always
   - ☐ Often
   - ☐ Sometimes
   - ☐ Rarely
   - ☐ Never
2. Do you wash your hands well after touching any part of your body (i.e., nose, mouth, face, hair) during food preparation or cooking?
   - ☐ Always
   - ☐ Often
   - ☐ Sometimes
   - ☐ Rarely
   - ☐ Never
3. Do you wash your hands well after touching raw meat?
   - ☐ Always
   - ☐ Often
   - ☐ Sometimes
   - ☐ Rarely
   - ☐ Never
4. Do you wash your hands well after touching raw eggs?
   - ☐ Always
   - ☐ Often
   - ☐ Sometimes
   - ☐ Rarely
   - ☐ Never
5. Do you wash your hands well during salad preparation?
   - ☐ Always
   - ☐ Often
   - ☐ Sometimes
   - ☐ Rarely
   - ☐ Never
6. Do you wash your hands well after coughing and sneezing during food preparation or cooking?
   - ☐ Always
   - ☐ Often
   - ☐ Sometimes
   - ☐ Rarely

- ☐ Never

7. Do you wash your hands well after disposing waste?
   - ☐ Always
   - ☐ Often
   - ☐ Sometimes
   - ☐ Rarely
   - ☐ Never
8. How much time do you spend on washing your hands?
   - ☐ 10 seconds or less
   - ☐ 11 to 19 seconds
   - ☐ 20 seconds or more
   - ☐ Depends on last thing I touched
   - ☐ Not sure
9. Do you wear gloves during food preparation or cooking?
   - ☐ Always
   - ☐ Often
   - ☐ Sometimes
   - ☐ Rarely
   - ☐ Never
10. Do you cover or tie your hair during food preparation or cooking?
    - ☐ Always
    - ☐ Often
    - ☐ Sometimes
    - ☐ Rarely
    - ☐ Never

COVID-19 questions

1. Do you disinfect the package of food delivered to your place before eating or storing it?
   - ☐ Always
   - ☐ Often
   - ☐ Sometimes
   - ☐ Rarely
   - ☐ Never
2. Do you wipe food packages after shopping with disinfectant (at home)?
   - ☐ Always
   - ☐ Often
   - ☐ Sometimes
   - ☐ Rarely
   - ☐ Never
3. Are you concerned of getting infected by the COVID-19 virus from eating food at restaurants?
   - ☐ Strongly disagree
   - ☐ Disagree
   - ☐ Neutral
   - ☐ Agree
   - ☐ Strongly agree

4. Are you concerned of getting infected by the COVID-19 virus from delivered food?
   - ☐ Strongly disagree
   - ☐ Disagree
   - ☐ Neutral
   - ☐ Agree
   - ☐ Strongly agree
5. Did the COVID-19 pandemic increase your concern on food safety?
   - ☐ Strongly disagree
   - ☐ Disagree
   - ☐ Neutral
   - ☐ Agree
   - ☐ Strongly agree
6. Did the COVID-19 pandemic increase your use of cleaning and disinfecting agents?
   - ☐ Strongly disagree
   - ☐ Disagree
   - ☐ Neutral
   - ☐ Agree
   - ☐ Strongly agree
7. During the COVID-19 pandemic, do you eat less from restaurants?
   - ☐ Strongly disagree
   - ☐ Disagree
   - ☐ Neutral
   - ☐ Agree
   - ☐ Strongly agree
8. Did the COVID-19 pandemic enhance your food safety measures in the following aspects?

	**Strongly disagree**	**Disagree**	**Neutral**	**Agree**	**Strongly agree**
Maintaining your personal hygiene at high level	☐	☐	☐	☐	☐
Controlling the temperature of food during storage, cooking and holding	☐	☐	☐	☐	☐
Cleaning and sanitation of food and food contact surfaces	☐	☐	☐	☐	☐
Decreasing food contamination	☐	☐	☐	☐	☐

<u>Cross contamination and Food consumption and safety</u>

1. How often do you store raw eggs separately (protected) from other foodstuffs in a refrigerator?
   - ☐ Always
   - ☐ Often
   - ☐ Sometimes
   - ☐ Rarely
   - ☐ Never

2. Do you eat fried or boiled eggs with running yolks?
   - ☐ Always
   - ☐ Often
   - ☐ Sometimes
   - ☐ Rarely
   - ☐ Never

Foodborne diseases

1. Do you think food poisoning is more serious for pregnant women?
   - ☐ Strongly disagree
   - ☐ Disagree
   - ☐ Neutral
   - ☐ Agree
   - ☐ Strongly agree
2. Do you eat pastries and cakes containing raw eggs (e.g., tiramisu)?
   - ☐ Always
   - ☐ Often
   - ☐ Sometimes
   - ☐ Rarely
   - ☐ Never
3. How frequently do you eat the following foods?

	**Never**	**Daily**	**2-3 times/week**	**1 time/week**	**Every two weeks**
**Cold deli meat**	☐	☐	☐	☐	☐
**White cheeses (Nabulsi cheese, Halloum, ect)**	☐	☐	☐	☐	☐
**Hot dogs**	☐	☐	☐	☐	☐
**Shawarma**	☐	☐	☐	☐	☐
**Raw fish (e.g., sushi)**	☐	☐	☐	☐	☐
**Fruits and vegetables without washing**	☐	☐	☐	☐	☐

Part 2: Knowledge

This part is designed to assess your knowledge about food safety. Please select the correct answer:

Personal Hygiene

1. Which of the following is the correct way to wash hands?
   - ☐ Wash with running cold water, wipe dry
   - ☐ Wash with running warm water, wipe dry
   - ☐ Use soap and then wash with running warm water, wipe dry
   - ☐ Wet hands with running warm water, use soap and then wash with running warm water, wipe dry
   - ☐ Whatever, it does not matter

Cross contamination

1. What will you do, if you cut raw meat with knife and want to use the same knife to chop vegetables? (choose the correct practice)
   - ☐ Use the same knife without washing
   - ☐ Wash it with water only
   - ☐ Wash it with water and soap
   - ☐ Wash it with water, soap and use disinfectant
   - ☐ Wipe it with a dry rag
2. If you put the raw meat and poultry in the middle shelf of refrigerator, then where do you put the vegetables and salads? (choose the correct practice)
   - ☐ In the Upper shelf of refrigerator
   - ☐ In the Middle shelf of refrigerator
   - ☐ Lower shelf of refrigerator
   - ☐ Whenever, does not matter
   - ☐ I do not know
3. The least safe way of tasting the food during cooking?
   - ☐ Put small quantity of food in the bowl and taste it
   - ☐ Use a clean spoon
   - ☐ Use the same spoon used to stir the food
   - ☐ It depends on the type of the food
   - ☐ I do not know

Temperature control

1. Which of the following is the safest way to defrost frozen raw meat or chicken? (choose the correct practice)
   - ☐ Leave it in kitchen table until it defrosts
   - ☐ leave it in refrigerator until it defrosts
   - ☐ Place it under running tap until it defrosts
   - ☐ Thaw it in microwave
   - ☐ Immerse it in frequently changed stagnant water
2. Which of the following is the correct way to heat leftovers? (choose the correct practice)
   - ☐ Heat it to the temperature you prefer
   - ☐ Reheat is not necessary if it's during the summer
   - ☐ Heat until it becomes warm
   - ☐ Heat until it boils
   - ☐ I do not know
3. How do you know that meat or poultry being cooked is already cooked? (choose the correct practice)
   - ☐ When water comes out of it
   - ☐ From its appearance
   - ☐ From its temperature
   - ☐ From experience
   - ☐ From touching

4. What should be done with prepared food that will be consumed 3 hours later? (choose the correct practice)
   - ☐ Put on a table in the kitchen
   - ☐ Cover it and put it on the cabinet
   - ☐ Put in the oven to keep warm
   - ☐ Put it in the refrigerator, then reheat when ready to eat
   - ☐ Cover it with blanket to keep it warm
5. What should be done if the reheated leftovers are still not eaten completely? (choose the correct practice)
   - ☐ Put in the refrigerator immediately and reheat before consuming
   - ☐ Store in kitchen and reheat before consuming
   - ☐ Store in kitchen and reheat before consuming as long as they smell good
   - ☐ Discard them immediately
   - ☐ I do not know
6. How long leftovers should be kept in the fridge? (choose the correct practice)
   - ☐ No more than 2 days
   - ☐ No more than 5 days
   - ☐ No more than 7 days
   - ☐ As long as the food has not gone bad
   - ☐ I do not know
7. When is the best time to purchase frozen food when shopping?
   - ☐ At the beginning of shopping time
   - ☐ In the middle of shopping time
   - ☐ At the end of shopping time
   - ☐ Whenever, does not matter
   - ☐ I do not know
8. At what temperature, do you think, chilled ready to eat foods should be kept?
   - ☐ Less than 1 ^{o}C
   - ☐ 1 to 4 ^{o}C
   - ☐ 5 to 10 ^{o}C
   - ☐ More than 10 ^{o}C
   - ☐ Not sure
9. At what temperature, do you think, frozen foods should be kept?
   - ☐ -18 ^{o}C
   - ☐ -8 ^{o}C
   - ☐ -4 ^{o}C
   - ☐ 0 ^{o}C
   - ☐ Not sure
10. At what temperature, do you think, hot ready to eat foods should be kept?
   - ☐ 21 to 30 ^{o}C
   - ☐ 31 to 40 ^{o}C
   - ☐ 41 to 50 ^{o}C
   - ☐ 51 to 60 ^{o}C
   - ☐ >60 ^{o}C
   - ☐ Not sure

11. At what temperature, do you think, chicken should reach during cooking?
    - ☐ 73ºC
    - ☐ 60ºC
    - ☐ 53ºC
    - ☐ Until it becomes warm
    - ☐ Not sure
12. How do you know food is contaminated with pathogenic bacteria (would make you sick)?
    - ☐ From smell
    - ☐ From taste
    - ☐ From color changing
    - ☐ It cannot be detected from its appearance or taste
    - ☐ Not sure

Cleaning and sanitation

1. The best way to avoid infections from fresh fruits and vegetables is to wash them with
    - ☐ Hot water
    - ☐ Anti-bacterial soap
    - ☐ Sponge and soap
    - ☐ Water mixed with one spoon of vinegar
    - ☐ Cold running water
2. Which of the following is the correct way to clean the kitchen countertop? (choose the correct practice)
    - ☐ Clean with a dry rag
    - ☐ Clean with a wet rag
    - ☐ Wash with a cleaning material, then wash it with water, then wipe with disinfectant
    - ☐ Clean it with water only
    - ☐ Whatever, it does not matter
3. To prevent food poisoning, how often should the kitchen sink drain in your home be sanitized?
    - ☐ Daily
    - ☐ Weekly
    - ☐ Monthly
    - ☐ Only when food is going to be thawed or washed in the sink
    - ☐ I do not know
4. The least safe method to disinfect the kitchen sponge
    - ☐ Soak the sponge with water and put it in microwave
    - ☐ Soak and wash in water
    - ☐ Soak in boiling water
    - ☐ Soak in water and chlorine
    - ☐ I do not know

Knowledge about Food Poisoning

Please answer True or False about the following signs and symptoms if they are related to food poisoning:

1. Diarrhea and vomiting	☐ True	☐ False
2. Abdominal pain and cramps	☐ True	☐ False
3. Hair falling	☐ True	☐ False
4. Hypertension (increase in blood pressure)	☐ True	☐ False
5. Drop in Blood Sugar	☐ True	☐ False
6. Cold and Cough	☐ True	☐ False

Knowledge about virus transmission

Please answer True or False about virus transmission in the following statements:

1. Women are at higher risk of complications than other healthy adults if they get Coronavirus.	☐ True	☐ False
2. COVID-19 virus can be transmitted by the food	☐ True	☐ False
3. COVID-19 virus can survive on hard surfaces for day(s)	☐ True	☐ False
4. COVID-19 virus can multiply in the food	☐ True	☐ False

Personal information

**1. What is your age in (Years)**	____________________						
**2. What is the number of pregnancies you had including the current one?**	______________________						
**3. How many months of pregnancy did you complete?**	____________________						
**4. What is your educational level**	○ Illiterate	○ Elementary education	○ Middle school	○ High school	○ Diploma	○ Bachelor's degree	○ Higher education
**5. Have you received any food safety information during pregnancy?**	○ Yes	○ No	○ Not Sure				
**6. If yes, please specify the source of information (choose all that applies)**	○ Doctor	○ Nurses	○ Nutritionist	○ Internet	○ Brochure	○ Family and friends	
	○ Television	○ Others					

Thank you for your participation!
